# Climate change and habitat fragmentation drive the occurrence of *Borrelia burgdorferi*, the agent of Lyme disease, at the northeastern limit of its distribution

**DOI:** 10.1111/eva.12165

**Published:** 2014-05-07

**Authors:** Julie A Simon, Robby R Marrotte, Nathalie Desrosiers, Jessica Fiset, Jorge Gaitan, Andrew Gonzalez, Jules K Koffi, Francois-Joseph Lapointe, Patrick A Leighton, Lindsay R Lindsay, Travis Logan, Francois Milord, Nicholas H Ogden, Anita Rogic, Emilie Roy-Dufresne, Daniel Suter, Nathalie Tessier, Virginie Millien

**Affiliations:** 1 Redpath Museum, McGill UniversityMontreal, QC, Canada; 2 Department of Biology, McGill UniversityMontreal, QC, Canada; 3 Ministère du Développement Durable, de l’Environnement, de la Faune et des Parcs duQuébec City, QC, Canada; 4 Département des Sciences Biologiques, Université de MontréalMontréal, QC, Canada; 5 Zoonoses Division, Centre for Food-Borne, Environmental & Zoonotic Infectious Diseases, Public Health Agency of CanadaSaint-Hyacinthe, QC, Canada; 6 Groupe de recherche en épidémiologie des zoonoses et santé publique Faculté de Médecine Vétérinaire, Université de MontréalSaint-Hyacinthe, QC, Canada; 7 Zoonoses & Special Pathogens Division, National Microbiology Laboratory, Public Health Agency of CanadaWinnipeg, MB, Canada; 8 Ouranos ConsortiumMontreal, QC, Canada; 9 Institut National de Santé Publique du QuébecLongueuil, QC, Canada

**Keywords:** climate change, emergence, habitat fragmentation, Lyme disease, range shift, white-footed mouse

## Abstract

Lyme borreliosis is rapidly emerging in Canada, and climate change is likely a key driver of the northern spread of the disease in North America. We used field and modeling approaches to predict the risk of occurrence of *Borrelia burgdorferi*, the bacteria causing Lyme disease in North America. We combined climatic and landscape variables to model the current and future (2050) potential distribution of the black-legged tick and the white-footed mouse at the northeastern range limit of Lyme disease and estimated a risk index for *B. burgdorferi* from these distributions. The risk index was mostly constrained by the distribution of the white-footed mouse, driven by winter climatic conditions. The next factor contributing to the risk index was the distribution of the black-legged tick, estimated from the temperature. Landscape variables such as forest habitat and connectivity contributed little to the risk index. We predict a further northern expansion of *B. burgdorferi* of approximately 250–500 km by 2050 – a rate of 3.5–11 km per year – and identify areas of rapid rise in the risk of occurrence of *B. burgdorferi*. Our results will improve understanding of the spread of Lyme disease and inform management strategies at the most northern limit of its distribution.

## Introduction

Climate change is thought to be driving geographic range shifts in many terrestrial species (Walther et al. 2002; Parmesan and Yohe 2003; Chen et al. 2011). In northern temperate regions especially, increasing temperatures are allowing species to expand their distribution poleward. Climate warming may thereby increase the opportunity for invasive species to establish and vector-borne diseases to emerge in new areas (Harvell et al. 2002; Wilcox and Gubler 2005; Jones et al. 2008). As global temperature increases, reservoir hosts and vector species may spread their habitats into more northern or southern latitudes and/or higher elevations (Zell 2004). Because the transmission of many parasites and pathogens depends on free-living wild animal reservoir hosts, a number of vector-borne infectious diseases have recently expanded their geographic range, tracking the range expansion of their hosts whose distribution is tied to climate (Ogden et al. 2006; Gage et al. 2008; Mills et al. 2010; Altizer et al. 2013).

Lyme borreliosis is a vector-borne disease caused by the spirochete *Borrelia burgdorferi* sensu lato and is an emerging disease in North America (Ogden et al. 2009; Ostfeld 2011). The main vector for Lyme disease in eastern and central North America is the black-legged tick *Ixodes scapularis* (Barbour and Fish 1993; Ostfeld 2011). The black-legged tick has a life cycle with three feeding stages. Small mammals and birds are hosts for the juvenile life stages of the tick (i.e., larvae and nymphs) and act as reservoir for the pathogen. The white-footed mouse (*Peromyscus leucopus*), a widespread rodent native of eastern North America (Desrosiers et al. 2002), is often the most common host for *I. scapularis* juvenile stages in woodlands of northeastern North America (Jones et al. 1998; Bouchard et al. 2011). The white-footed mouse is a highly efficient reservoir host for *B. burgdorferi*, being highly susceptible to infections (Shaw et al. 2003; Eisen et al. 2012) that are persistent (often lifelong) and transmissible with high efficiency to uninfected larval ticks (with up to 90% of larvae acquiring infection) that subsequently feed on the infected mouse (Donahue et al. 1987; Ostfeld 2011).

Both the white-footed mouse and the black-legged tick are essential components of the transmission cycle of *B. burgdorferi*. Historical records (Roy-Dufresne et al. 2013) and molecular evidence (unpublished data) show that the white-footed mouse is currently expanding its range poleward, presumably in response to milder winters (Myers et al. 2009; Roy-Dufresne et al. 2013). The black-legged tick is also expanding its geographic range northward, tracking climate warming over the last few decades (Ogden et al. 2006; Leighton et al. 2012).

As exemplified by the ongoing and concurrent shifts of the white-footed mouse and black-legged tick in Southern Québec, environmental change is considered as the main driver of the shifts in the distribution of vectors and hosts, and hence of disease emergence (Gould and Higgs 2009). Climate and the local habitat change could interact and affect the dispersal and movement of the species involved in the transmission cycle of Lyme disease in a complex manner. For example, a warmer climate with milder winters and earlier spring snowmelt may shift the phenology of the white-footed mouse breeding activity and dispersal, with higher activity and movement earlier in the season. Increased activity in turn alters the rate of encounter between this host and its pathogens, affecting the dynamics of transmission cycle of *B. burgdorferi* (Ogden et al. 2007, 2008a). On the other hand, fragmentation of host habitat may reduce host population size, limit host dispersal, and alter host densities and diversity (Daszac et al. 2001; Li et al. 2012), reducing encounter rate between hosts and pathogens and the transmission rate of the pathogen. Conversely, the spread of the pathogen may occur faster if range expansion occurs in host species such as the white-footed mouse that thrives in fragmented landscapes (Allan et al. 2003; Chen et al. 2011).

Disease occurrence is thus constrained by a number of interacting environmental factors. In addition, the different species involved in a multispecies host–pathogen system may not respond similarly to changes in the environment (Slenning 2010). Different species are expected to shift their ranges at different rates (Chen et al. 2011), depending on their physiological tolerance, life-history traits or dispersal ability (Altizer et al. 2013). Vector-borne disease occurs in environments where the geographic range of competent vectors and wildlife reservoir hosts overlap, and understanding the factors that constrain this region of overlap is thus key to predicting the effect of global changes on vector-borne diseases. Risk maps of population establishment for the black-legged tick in Canada have been published (Ogden et al. 2008b). These have been used as a proxy for Lyme disease risk because both *I. scapularis* and *B. burgdorferi* are host generalists. However, these models did not account for the shift in the distribution of the white-footed mouse in Eastern Canada. Yet, changes in *P. leucopus* host distribution and abundance could be key for determining the level of risk from *B. burgdorferi* because these mice are such efficient reservoir hosts.

Here, we use an integrated approach and combine climate niche modeling with landscape models to estimate the relative contributions of climate and habitat change on the distribution of two key components in the Lyme disease system in northeastern America, the white-footed mouse and the black-legged tick. Concurrent with recent and rapid climate warming, we expect a higher prevalence of *B. burgdorferi* in hosts and ticks in more disturbed, fragmented landscapes, which favors the white-footed mouse over other forest small mammals (Allan et al. 2003; Killilea et al. 2008). We predict that climate is limiting the most northern limit of expansion of *B. burgdorferi*, but that this expansion is locally modulated by landscape fragmentation. To test this hypothesis, we apply our model to predict the current and future distribution of *B. burgdorferi*. By combining both empirical data from multiple species and field sites, as well as modeling approaches, we aimed to better predict patterns of emergence and spread of Lyme disease at the most northern limit of its distribution in Southern Québec, to better inform management strategies in a zone of emergence of the disease.

## Materials and methods

We first used field data to identify the parameters related to the black-legged tick, the white-footed mouse and small mammals that best explained the observed pattern of *B. burgdorferi* occurrence at our study sites. We then modeled the relation between landscape variables and those variables for the white-footed mouse and the black-legged tick. Our landscape models were then combined with climate niche models for these species to estimate the risk of occurrence of *B. burgdorferi* over our study area. Finally, we extrapolated the risk index at a larger provincial scale, for today and the future (2050).

### Study sites

**Figure 1:**
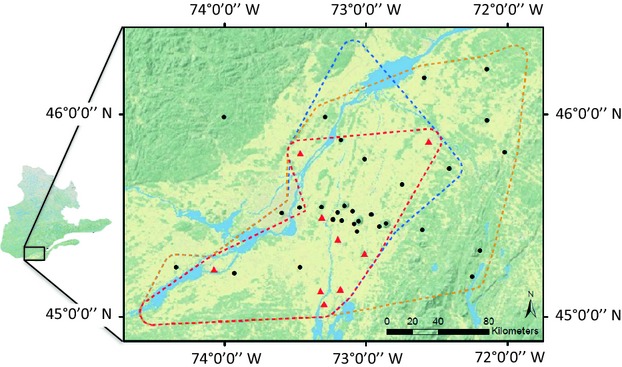
Study area and sampling site in Southern Québec, Canada, between 45.00–46.25°N and 72.00–74.50°W. The study extent is covered by 41.3% woodlands, 39.9% agriculture fields and 9.8% urban areas. Symbols for field sites: black circles = *Borrelia burgdorferi* absent, red triangle = *B. burgdorferi* present. The distribution limits of *Ixodes scapularis* (orange dotted line), *Peromyscus leucopus* (blue dotted line) and *B. burgdorferi* (red dotted line) were drawn using the ArcGIS toolbox with a buffer of 5 km around the study site where each was detected.

We surveyed a total of 34 sites in southern Québec from May to October 2011 (Fig. 1). The Système d’information écoforestière (SIEF) was used as a spatial representation of the study area (MRNF 2004). The study area covers 26 682 km^2^ and is characterized mostly by woodlands (41.28%), agricultural fields (39.94%) and urban areas (9.79%). The study sites were located in forest fragments of different size and degree of isolation within a matrix dominated by agricultural fields (Fig. 1). The study area included the most northern known distribution limit of the white-footed mouse and the black-legged tick in eastern Canada.

### Small mammal and tick sampling

Small mammals were trapped from June to early September. At each site, four grids of 28 Sherman™ live traps were placed for one night in four parallels transects of seven traps each separated by 10 m (thus forming 60 × 30 m grids). Trapping occurred for another two consecutive nights if no *Peromyscus* spp. mice were captured on the first night. All captured individuals were assigned to a species using a molecular method to differentiate *Peromyscus* species (Rogic et al. 2013).

Questing (host-seeking) ticks were collected from the vegetation using a standard drag sampling method (Falco and Fish 1992; Ostfeld et al. 1995). Each site was visited for drag sampling three times (spring, summer and fall); this frequency follows the recommendation of the Public Health Agency of Canada for the surveillance of tick populations in the country. This sampling accounts for the seasonality of adult, nymphal and larval ticks, which occurs at different seasons.

In the spring (May–June) and fall (September–October) visits, the sampling occurred over an area of 105 × 500 m. We established a set of four parallel transects of 500 m and 25–35 m apart. Ticks were collected from the drag every 25 m. During the summer (July–August), questing ticks were sampled by dragging over the small mammals trapping grids. Feeding ticks were collected directly from small mammals examined under the microscope. All ticks sampled were assigned to a species on examination in the laboratory using standard keys (Keirans et al. 1996) and to one of the three life stages (i.e., larval, nymph, or adult).

### Detection of *Borrelia burgdorferi*

All mammals, questing ticks, and feeding ticks collected were tested for the presence of *B. burgdorferi* following a method described in Ogden et al. (2011). Briefly, DNA was extracted from ticks and mammal tissues (heart) and screened for *B. burgdorferi* with a multiplex real-time polymerase chain reaction (PCR) targeting the 23s rRNA of *B. burgdorferi*. We screened the heart of small mammals as it has been shown to be a reliable tissue to target for the detection of systemic infection of individuals (Barthold et al. 1993; Baum et al. 2012). *Borrelia burgdorferi* infection was confirmed in positive samples using a *B. burgdorferi* specific primer targeting the ospA gene (Ogden et al. 2011). All PCR assays were performed at the National Microbiology Laboratory of the Public Health Agency (Winnipeg, MB, Canada).

### Modeling the relations between small mammals, ticks and *Borrelia burgdorferi* distribution patterns

We used our field data to investigate the relationships between the presence of *B. burgdorferi* at a site and a number of variables related to ticks and small mammals, specifically the white-footed mouse *P. leucopus*. For ticks, we considered the number of questing ticks (all life stage included), the number of feeding larvae, and the total number of ticks (i.e., both questing and feeding on small mammals, all life stages included). We then calculated the prevalence of small mammals infested with ticks (the number of individual small mammal carrying ticks per total number of individual small mammal captured at a site), as well as the mean tick infestation of small mammals at a site (the mean number of ticks feeding per small mammal individual captured). Values for infestation prevalence and infestation levels were estimated for *P. leucopus* alone, and for all other small mammals collectively. We also considered the density of small mammals, the density of *P. leucopus*, small mammal diversity (estimated with the Shannon index), species richness (the total number of small mammal species at a site), and the proportion of *P. leucopus* in the community relative to other small mammal species. As trapping effort varied across site due to unequal number of trap nights, we estimated small mammal and *P. leucopu*s densities as the mean number of individuals captured per night per square km. Although likely a biased estimate of the actual density of small mammals at our trapping sites, this estimate was corrected for trapping effort in order to be comparable across sites. We also corrected the number of feeding ticks by the number of trapping night at each site by dividing the number of ticks by the number of trapping nights.

Some of the variables were correlated with each other, and thus, we performed a hierarchical partitioning analysis using the hier.part package In R (Walsh and MacNally 2013) to estimate their contribution to the total variance, both independently and jointly (MacNally 2000, 2002). We used a logistic regression model with all our field data as explanatory variables, and the presence of *B. burgdorferi* at a site as the dependent variable. We evaluated the significance level of the independent contribution of each variable to the variance in the model with a randomization test (1000 permutations), using the rand.hp function in the hier.part package in R.

### Predicting the distribution of the black-legged tick and the white-footed mouse from the climate

#### Black-legged tick

Temperature has been described as a good predictor of the abundance of the black-legged tick *I. scapularis* at the northern limit of its geographic range in Canada (Ogden et al. 2005, 2006, 2008b). We used the linear relation between the maximum number of adult female ticks, an index of tick abundance, and the annual cumulative degree-day (DD) > 0°C (NumberT = 0.436 × DD > 0–1232; Ogden et al. 2005) to estimate the abundance of ticks over our study area. DD > 0 data were averaged over 1961–2005 and were derived from the ANUSPLIN dataset version 4.3 (The Australian National University, Canberra, Australia) based on Environment Canada’s historical monthly ~10 × 10 km gridded weather data covering the entire territory of Québec (McKenney et al. 2006). Tick abundance was considered null when DD was lower than 2800 (Ogden et al. 2005). This model was validated by testing the fit between the predicted abundance from the model and the total tick abundance measured at our study sites, using a Spearman’s rank correlation coefficient. We used the observed total abundance of ticks of all developmental stages rather than just the number of adult females. This was because these values were correlated but also because adult ticks are the least abundant instar (with high levels of mortality) and are at particularly low density at sites where the ticks have only recently become established (Ogden et al. 2010). The use of adult ticks alone would have resulted in some sites having zero values for ticks, when in fact ticks were present.

#### White-footed mouse

We used the predictions from a climate niche model for the white-footed mouse based on a combination of climatic variables related to winter conditions (Roy-Dufresne et al. 2013). The white-footed mouse appeared to be primarily limited by the winter length and average maximum temperature in the winter, the probability of occurrence of the mouse decreasing with longer (>125 to 160 days) and colder (maximal temperature <−5°C) winters. We used the occurrence probability from this model projected at a resolution of 10 km^2^, the same resolution we used to predict tick abundance over our study area. A Spearman’s rank correlation coefficient was then used to test the fit between the predicted presence probability of *P. leucopus* and density observed at our study sites for this species.

### Predicting the distribution of the blacklegged tick and the white-footed mouse from the landscape

We used the Data Management Tool in ArcGis 10.1 (ESRI 2013) to estimate the proportion of woodlands, agricultural fields and urban areas around each of the 34 study sites within a buffer of 5 km, a distance much larger than reported natal dispersal distances of the white-footed mouse (~100 m on average, Keane 1990). The mean landscape resistance—a proxy for the impediments to the migration of individuals between breeding sites—was then estimated around each forest patch, using values from R. R. Marrotte, A. Gonzalez, and V. Millien (unpublished data). A patch was defined in the study area as a forested area surrounded by nonforested areas. The genetic differentiation between 11 populations of the white-footed mouse found in forest patches within our study area was previously estimated in Rogic et al. (2013) using 11 microsatellite loci. We then used these results to estimate resistance values for the three main landcover categories (R. R. Marrotte, A. Gonzalez, and V. Millien unpublished data). Woodlands were assigned a resistance of 0 (no resistance), while the resistance was 0.49 for agricultural fields and 4 for urban areas. Using the same method, we then calculated the mean resistance for all (9043) forest patches in our study area. We extracted the area, perimeter, and minimum distance to the nearest neighbor (MDNN) calculated between the centroids of each forest patch. Lastly, we estimated the connectivity of all 9043 forest patches using the one to all modeling mode and an eight neighbors and average resistance connection scheme in CircuitScape (McRae 2006). We aggregated the SIEF data into five classes (agriculture, forest, orchard, urban, water and other, a class which is an aggregate of the remaining land uses). We then rasterized this spatial data to a resolution of 300 m using the raster package in R (Hijmans & vanEtten 2012) and assigned circuit resistance values from R. R. Marrotte, A. Gonzalez, and V. Millien (unpublished data) to each class. Finally, we used the conductance, or the inverse of the estimated resistance, as an estimate of connectivity.

We performed a principal component analysis (PCA) on landscape variables at our study sites (proportion of forest, agriculture and urban habitat, area, perimeter, MDNN, resistance, and connectivity). The fit between the white-footed mouse density and the total tick abundance measured at a site and the loading of this site on the first three axes of the PCA was evaluated with a Spearman’s rank correlation test. We then used a linear model to predict the white-footed mouse density at a site from the PCA axes with which mouse density was significantly correlated. Similarly, we used a generalized linear model with a negative-binomial distribution to predict tick abundance at a site from the PCA axes with which tick abundance was significantly correlated. Next, we measured our landscape variables within a 5 km buffer for each of the 9043 forest patches in our study area. Using the models described above between mouse density, tick abundance, and the PCA axes, we then estimated the coordinates of these patches on the first two PCA axes from these landscape variables and calculated the predicted density of white-footed mouse and maximum abundance of black-legged tick for each of these patches. Finally, both the mouse density and the tick abundance were extrapolated over the entire study area by calculating the mean value for all forest patches in each pixel of 10 km × 10 km on a grid of 1052 cells.

### Predicting the current distribution of *Borrelia burgdorferi*

We calculated the risk index for *B. burgdorferi* presence as a linear combination of the four factors described above: (i) the predicted tick abundance from the climate, (ii) the predicted *P. leucopus* presence probabilities from climatic variables, (iii) the predicted *P. leucopus* density from the landscape variables, and (iv) the predicted tick abundance from the landscape variables. To do so, we used a hierarchical variance partitioning analysis to assess the variance contribution of each of these four factors (MacNally 2000, 2002). We used the percentage of variance explained by each factor (independent contribution) as a coefficient of importance in the final linear model. At the local scale, we used the four factors to estimate the risk index and the resulting risk index value was rescaled to range from 0 to 1. At the regional scale, only the first two factors were used to estimate the risk index. This risk index provides a relative estimate of the risk that can be used to compare risk among different locations.

### Predicting the future distribution of *Borrelia burgdorferi*

Future climate scenarios for 2050 were created using simulated future climate data from the Canadian Regional Climate Model (CRCM version 4.2.3) (Music and Caya 2007) and an ensemble of global climate simulations from the Coupled Model Intercomparison Project (CMIP3) (Meehl et al. 2007). As in Roy-Dufresne et al. (2013), we used a total of 37 simulations divided among three SRES emissions scenarios (12 A1b, 15 A2, and 10 B1; Nakicenovic et al. 2000).

The projected future distribution and abundance of *I. scapularis* in Quebec was estimated from the linear model linking DD > 0 and maximum tick abundance published by Ogden et al. (2005). The future distribution of the white-footed mouse was obtained from the projected presence probabilities in Roy-Dufresne et al. (2013). We kept the landscape constant for future projections and estimated the future risk index for the presence of *B. burgdorferi* in Southern Québec by 2050, based on future scenarios of climate change and associated future distributions of the black-legged tick and white-footed mouse.

## Results

### Wild small mammals trapping and tick collection

We captured a total of 520 small mammals at the 34 sites representing six different species, and species richness at a site ranged from one to four species (Table S1). *Peromyscus leucopus* was the most common species (*n* = 312) and was present at 24 sites with a mean capture rate (i.e., number of captured *P. leucopus*/total number of captured mammals) of 60%. Other species captured were the deer mouse (*Peromyscus maniculatus*, *n* = 60), the red-backed vole (*Myodes gapperi*, *n* = 62), the short-tailed shrew (*Blarina brevicauda*, *n* = 43), smoky and/or masked shrews (*Sorex* sp., *n* = 33) and the woodland jumping mouse (*Napaeozapus insignis*, *n* = 10), with capture rates of 11.5%, 11.9%, 8.3%, 6.3%, and 1.9%, respectively.

We collected a total of 1417 ticks of seven species from the vegetation and from small mammals at 31 of our 34 field sites. *Ixodes scapularis* was the most common species (*n* = 1130), followed by *Dermacentor albipictus* (*n* = 264), *Haemaphysalis leporispalustris* (*n* = 12), *Ixodes angustus* (*n* = 7), *Ixodes marxi* (*n* = 2), *Ixodes muris* (*n* = 1), and *Ixodes cookei* (*n* = 1). We collected a total of 311 *I. scapularis* feeding on hosts at 20 sites. A vast majority (*n* = 724) of *I. scapularis* were larvae (429 questing and 295 feeding on small mammals), 311 were nymphs (295 questing and 16 feeding) and 95 were adults (all questing). An exceptionally high number of *I. scapularis* was collected from one site (*n* = 504), and the number of collected ticks ranged from 1 to 137 in the other 30 sites where ticks where detected.

On average, *P. leucopus* tended to carry ticks more often than other small mammals (average Prevalence L = 16.67 vs average Prevalence M = 6.34, Wilcoxon rank test, *W* = −431.5, *P* < 0.04), but when carrying ticks, *P. leucopus* did not carry a larger number of ticks than other small mammals (*P* = 0.14).

### *Borrelia burgdorferi* prevalence

*Borrelia burgdorferi* was detected at nine of our study sites (Fig. 1, Table S1). Five mammals (four *P. leucopus* and one *P. maniculatus*) tested positive for *B. burgdorferi*. These five mice all carried feeding *I. scapularis* larvae and only one of those was positive for *B. burgdorferi*.

Fifty-three of the 390 questing *I. scapularis* captured in the vegetation and screened for the bacterium (questing larvae were not processed) tested positive for *B. burgdorferi*, an infection rate of 13.6% (23.2% in adults and 10.2% in nymphs). Of the 311 *I. scapularis* sampled on small mammals and screened for *B. burgdorferi*, only two tested positive. These two ticks were larvae feeding on two individuals of *P. leucopus*.

### Small mammals and tick distribution patterns and occurrence of *Borrelia burgdorferi*

There was no spatial autocorrelation between any of the small mammal, ticks or habitat variables in our dataset (*z* Moran’s index ranging from −0.03 to 0.94, all *P* > 0.35, with a buffer of 66.32 km corresponding to the minimal Euclidean threshold distance for which each site had at least one neighbor), and thus, all variables were considered statistically independent.

We performed a hierarchical partitioning analysis considering a total of 12 explanatory tick, mouse and small mammal variables we measured in the field. Three variables contributed most to the model predicting the presence of *B. burgdorferi* at a site (Table 1): the proportion of *P. leucopus* carrying ticks (PrevalenceL, independent contribution to the variance *I* = 20.06%, *P* < 0.05), the total number of ticks (Total T, *I* = 14.86%, *P* < 0.05) and the number of questing ticks (QuestingT, independent contribution to the variance *I* = 13.30%, *P* < 0.05). This result is in line with our observation that the presence of *B. burgdorferi* was most likely at sites where both the white-footed mouse and the blacklegged tick co-occurred (Fig. 1).

**Table 1:** Explanatory variables ranked according to their independent effect. I is the percentage of explained variance accounted for by the variable calculated using a hierarchical partitioning analysis performed with the tick, small mammals and *Peromyscus leucopus* variables as explanatory variables and the probability of occurrence of *Borrelia burgdorferi* as the response variable.

Variable	I (%)	*Z*-score
**Prevalence L**	20.06	7.83*
**Total T**	14.86	4.13*
**Questing T**	13.30	3.95*
**Feeding L**	9.75	2.46*
**Prevalence M**	9.56	3.25*
**Burden L**	6.79	1.81*
Diversity	5.61	1.30
Burden M	4.49	0.64
Proportion	4.36	0.78
Density M	4.24	0.78
Richness	3.90	0.55
Density L	3.06	0.13

Prevalence L: number of individual *P. leucopus* carrying ticks per total number of individual small mammal captured at a site, Total T: Number of ticks collected at a site both in the vegetation and on small mammals and of all life stages, Questing T: Number of ticks of all life stages collected in the vegetation at a site, Feeding L: Number of larvae collected on small mammals at a site divided by the number of trap nights, Prevalence M: number of individual small mammal carrying ticks per total number of individual small mammal captured at a site, Burden L: mean number of ticks feeding per *P. leucopus* individual captured at a site, Diversity: small mammal diversity at a site estimated with the Shannon index, Burden M: mean number of ticks feeding per small mammal individual captured at a site, Proportion: proportion of *P. leucopus* in the community relative to other small mammal species, Density M: mean number of small mammal individuals captured at a site per night per unit area (1 km^2^), Richness: total number of small mammal species at a site, Density L: mean number of *P. leucopus* individuals captured at a site per night per unit area (1 km^2^).

*Z*-scores were calculated to estimate the significance level of I for each explanatory variable, using 1000 randomizations and significant variables are in bold (*P < 0.05).

### Predicted distribution of the blacklegged tick and the white-footed mouse

#### Climatic models

**Figure 2:**
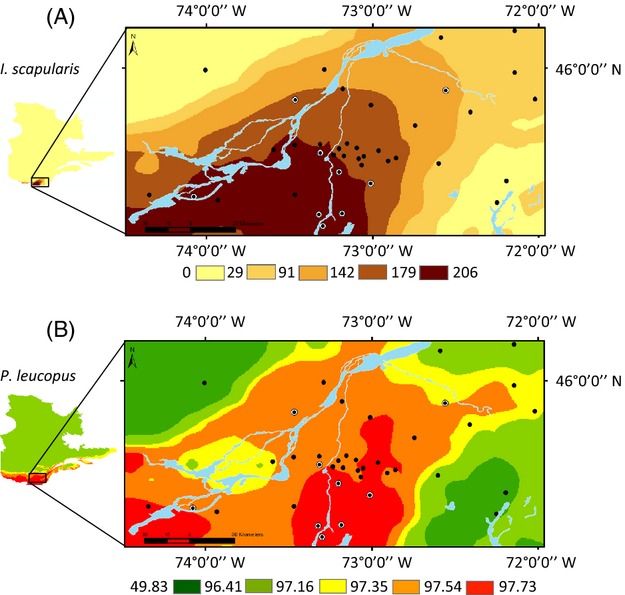
Current predicted distribution of the black-legged tick, that is, the maximum annual number of feeding female ticks at equilibrium (A) and the probability of presence of the white-footed mouse (B) within the study area, based on climatic variables. On both panels, filled black circles are field sites where *Borrelia burgdorferi* was not detected and black circles with a white outline are field sites where *B. burgdorferi* was detected.

The maximum annual number of feeding female ticks at equilibrium predicted by the current average DDs > 0°C ranged from 0 to 206 over the study area (Fig. 2A) and in Québec (Figure S1A). Tick abundance gradually decreases from the southwestern to the northeastern part of the region. Higher abundances are observed south of the Saint-Lawrence River and extend from the border with the United States to the south, to the northwestern limit of the Montérégie. The predicted abundance of ticks was the lowest in the Estrie region, and intermediate in the Centre du Québec region (Fig. 2A). These predicted values were correlated with the total tick abundance observed at our sites (*ρ* = 0.45, *P* < 0.007). Using this model between DDs > 0°C and tick abundance, the distribution of the tick is expected to shift northward by up to 300 km by 2050 (Figure S1B).

The predicted probability of presence of the white-footed mouse obtained from the climate distribution model ranged from 49.83% to 97.51% over the study area (Fig. 2B) and rapidly decreased above 45°N (Figure S2A). Two areas appeared to be highly suitable for the establishment of *P. leucopus*: the center of the Montérégie and the South of the Saint-Lawrence River to the southwestern tip of the Montérégie, where presence probabilities were >80%. The current predicted presence probability of the white-footed mouse was significantly correlated with the mouse density observed at our study sites (*ρ* = 0.45, *P* < 0.007). By 2050, the distribution of the white-footed mouse is predicted to shift poleward by approximately 250 km (Figure S2B, Roy-Dufresne et al. 2013).

#### Landscape models

The first two components of the PCA performed on the landscape variables explained a cumulative variance of 73.07% (Figure S3). The first axis represented a landscape gradient from agricultural to woodland, with increasing forest patch size and edge habitat and decreasing degree of fragmentation. It was positively correlated with the proportion of woodlands (*r* = 0.96), the forest patch area (*r* = 0.94) and the forest patch perimeter (*r* = 0.94), and was negatively correlated with the proportion of agricultural fields (*r* = −0.76). The second axis was positively correlated with the proportion of urban areas (*r* = 0.95) and the mean resistance (*r* = 0.87), and negatively correlated with connectivity (*r* = −0.51) and the proportion of agriculture fields (*r* = −0.52). The minimal distance to the nearest neighbor was correlated with the third component (*r* = 0.98), which further explained 12.59% of the variance.

There was a negative relation between the first PCA axis and tick abundance and we used a negative-binomial model to predict tick abundance (Total T = 2.55–0.37 PC1, Residual deviance = 39.05, *P*(estimate) < 0.004).

The observed density of white-footed mice was negatively correlated with the first axis (*ρ* = −0.55, *P* < 0.0008). We used the first axis of the PCA to predict the density of the white-footed mouse, using a linear model (Density L = 0.41–0.10 PC1; *R*^2^ = 0.24, *F* = 10.29, *P* < 0.003). Both the density of the white-footed mouse and the abundance of ticks were larger in a fragmented landscape of small forest patches within an agricultural matrix and decreased in areas predominantly covered with large continuous forest patches.

### Current distribution of *Borrelia burgdorferi*

We used a combination of our four factors to model the risk of occurrence of *B. burgdorferi*: (i) the predicted maximum abundance of ticks obtained from DD > 0, (ii) the presence probability of the white-footed mouse obtained from the climate distribution modeling, (iii) the estimated white-footed mouse density predicted from the first PCA axis of the landscape variables, and (iv) the estimated tick abundance from the first PCA axis of the landscape variables.

These four variables were correlated with each other (all *ρ* > 0.42 and all *P* < 0.012), and we used a hierarchical partitioning analysis to evaluate both the independent and joint contribution of the four variables on the presence of *B. burgdorferi*. The independent contribution of *P. leucopus* presence probability predicted by the climate was the largest (64.32%, *z* = 4.33, *P* < 0.05). The independent contribution of the tick abundance by the climate was 18.96%, the independent contribution of *P. leucopus* densities predicted by the landscape was 8.38% and the contribution of the tick abundance from the landscape was 8.35%, but they were not significant. We nevertheless kept all the four factors in our model, as they also contributed jointly to the variance in the risk of occurrence of *B. burgdorferi* at a site, a contribution whose significance level cannot be estimated with the analysis.

Next, we used these coefficients to estimate a risk index for the presence of *B. burgdorferi* over the study area. We used the following formula to calculate this index:

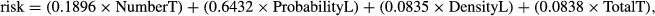


where Number T is the tick abundance (maximum number of feeding females) estimated from the mean annual DD, Probability L is the presence probability of the mouse estimated from the climate niche model, Density L is the density of the mouse at a site estimated from the landscape variables and Total T is the tick abundance estimated from the landscape variables. The risk index was considered null when the estimated tick abundance Number T = 0.

**Figure 3:**
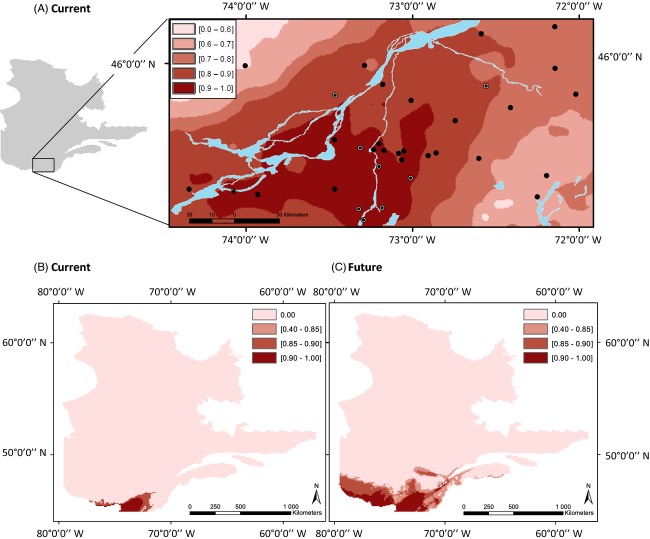
Current (A, B) and future (2050) (C) risk index for the presence of *Borrelia burgdorferi*. The future risk was estimated for a change in climate under a combination of A2, A1b, and B1 greenhouse gas emissions scenarios from the IPCC (Nakicenovic et al. 2000) (WGS 1984 World Mercator). The risk index at the local scale (panel A) was estimated from the distribution patterns of the tick and the mouse with both climatic and landscape variables. The risk index at the regional scale (panels B and C) was estimated from the distribution patterns of the mouse and the tick with climatic variables only. Both risk indices have been bounded from 0 to 1 and are relative estimates of the risk within a region that can be used to compare the relative risk between two specific areas within the region. The risk maps were generated with risk index classified into quintiles.

The risk index was null at one of the field sites and ranged from 0.63 to 0.97 at the other 33 study sites (Fig. 3A). A higher risk of presence was predicted in the southwestern part of the Montérégie, rapidly decreasing from the Canadian border to the northwestern end of the Montérégie (Fig. 3A). All nine positives sites, where *B. burgdorferi* was detected in the field, were located in an area of higher predicted risk (>0.87).

Lastly, we estimated the risk index at the scale of Québec, without taking into account the effect of the landscape on the density of the mouse or the abundance of the ticks, since landscape variables were not available at this large geographical scale. We performed a hierarchical partitioning analysis considering only the distribution of the mouse and the tick estimated from the climate and found that independent contributions of the mouse probability of occurrence and the tick abundance to the variance in the presence of *B. burgdorferi* were both significant. The probability of presence of the mouse obtained from the climate niche modeling explained 69.60% of the variance (*z* = 4.07, *P* < 0.05), and the maximum tick abundance estimated from the DD > 0 was 30.40% (*z* = 1.73, *P* < 0.05). The risk index for *B. burgdorferi* was thus as follow: risk QC = (0.3040 × Number T) + (0.6960 × Probability L). The risk index was considered null when Number T = 0. At this scale, the maximum risk index was 0.74 and rapidly decreased with latitude (Fig. 3B). The risk index was null across most of the province of Quebec.

### Future *Borrelia burgdorferi* distribution

We predict a shift of approximately 1.5 degrees of latitude or 150 km north of the zone of high risk (risk index >0.65) by 2050 (Fig. 3C) in Southern Québec. This represents a rate of northern expansion of approximately 3.5 km per year. A greater shift is predicted to occur in the northeastern direction, along the shores of the St Lawrence River, where zones with a risk index >0.65 are predicted to shift by 450 km, at a rate of approximately 11 km per year. Another region of greater increase in the risk index is along the shores of the Saguenay River. Regions above 49 degree latitude will not likely be exposed to *B. burgdorferi* by 2050.

## Discussion

We predict a northward shift of *B. burgdorferi* into Southern Québec at a rate of approximately 3.5–11 km per year over the next 40 years. Using empirical evidence and data collected in the field, we provide a first quantitative estimate of the relative importance of climate versus landscape variables on the distribution of the white-footed mouse and the black-legged tick. We combined these to estimate a risk index for the presence of *B. burgdorferi* hot spots. The environmental risk for Lyme disease, estimated from the abundance of ticks, the presence of *B. burgdorferi* and the proportion of ticks infected at a site (Tsao 2009), is a strong predictor of human Lyme disease case incidence in the USA (Pepin et al. 2012). Here, we show that in our study area this risk is also dependent on the distribution and abundance of the white-footed mouse, a rapidly expanding species in southern Québec. Our results therefore provide essential information for efforts to anticipate and manage the spread of Lyme disease in this region of emergence.

### Current status of Lyme borreliosis and active surveillance in Québec

The first human case of Lyme disease acquired in Québec was reported in 2008 (Milord et al. 2013; INSPQ 2013). The number of cases reported in the province has increased from 2 in 2004 to 112 in 2013 (incomplete data for 2013, Jodoin et al. 2013); of the 245 cases reported in Québec since 2004, 80 were indigenous (49 in 2013). Based on this evidence for an increase in Lyme disease in Southern Québec, public health agencies recommended the continuation of active surveillance in the region (Milord et al. 2013). Active surveillance in Québec has been focused on the detection of established tick populations in the province (e.g. Ogden et al. 2010), as well as identifying the presence *B. burgdorferi* and the prevalence of infection in ticks toward a better understanding of environmental risk for the pathogen. With the assumption that there is a link between the abundance and spread of the black-legged tick and the occurrence of Lyme disease in humans, some have attempted to predict the risk of *B. burgdorferi* estimated from tick distribution and abundance (e.g. Glass et al. 1995; Kitron and Kazmierczak 1997). These models however, did not take into account other key agents in the transmission cycle of Lyme disease.

### The importance of the white-footed mouse and black-legged ticks

The transmission cycle of *B. burgdorferi* is complex, and involves a suite of obligatory hosts for the vector tick, for which suitable habitat and environmental conditions must exist. Ticks hatch from eggs as larvae, then develop into nymphs and finally adults over a two and a half- to 3-year life cycle. At each transition from one life stage to the next, they require a single blood meal taken from a host. *B. burgdorferi* transmission cycles involve acquisition of infection from an infected host by a larval tick, maintenance of infection through development and molting to a host seeking nymph, and transmission of infection to a new host by the infected nymph. The efficiency of the transmission cycle in a given location is then determined by the relationships between the tick and its stage-specific host, in particular by the proportion of ticks that feed on competent reservoir hosts, and the efficiency of the competent reservoir host species to transmit the infection to feeding ticks. In northeastern USA, the white-footed mouse is a very common host for *I. scapularis* larvae and is also a highly efficient reservoir for *B. burgdorferi*, infecting 75–90% of the larvae it feeds, and maintaining infection lifelong (Donahue et al. 1987; Ostfeld 2011). We thus expect the white-footed mouse to be highly influential in the efficiency of cycles of *B. burgdorferi* transmission among different vertebrate reservoir hosts and in the proportion of host-seeking ticks in any one location that are infected. The white-footed mouse can therefore be key in determining Lyme disease risk for humans.

Leighton et al. (2012) estimated a range expansion of 46 km per year over the next decade for *I. scapularis* in Canada. The predicted rate of expansion for the white-footed mouse is lower, at an estimated 15 km per year in Michigan (Myers et al. 2009) and 10 km per year in Southern Québec (Roy-Dufresne et al. 2013). We attribute this difference to the difference in dispersal behavior of the two species. Ticks benefit from long-distance dispersal by migratory birds (Ogden et al. 2008c), which may be responsible for the rapid range expansion estimated by Leighton et al. (2012). Yet, while migratory birds disperse ticks from populations in the US into southern Canada, they mostly disperse ticks during the spring at the peak of activity of nymphal stage (Ogden et al. 2008c; Brinkerhoff et al. 2011). These nymphs then molt into adults that then feed on white-tailed deer, which are dead-end hosts for *B. burgdorferi*. Furthermore, the environmental conditions at most northern locations where ticks are dropped may preclude the establishment of reproducing tick population at these sites. As long as the habitat is suitable (i.e. it provides a duff layer that acts as a refuge protecting ticks from far subzero temperatures), *I. scapularis* readily survive over winter even when air temperatures reach −30°C, so minimum winter temperatures have a limited impact on where *I. scapularis* populations can establish (Lindsay et al. 1995; Brunner et al. 2012). However if spring, summer, and autumn temperatures are too low, the temperature-dependent duration of development from one life stage to another becomes too long and the probability that a larva survives to be a reproducing adult falls below unity beyond a certain northern latitudinal limit (Ogden et al. 2005). So while ticks are potentially able to disperse over large distances, several hundreds of kilometers, the local environmental conditions constitute a hard boundary for their development and survival. Mice, on the other hand, rely solely on their own dispersal ability to shift their range. We found that the presence of *B. burgdorferi* at a site was related to the proportion of white-footed mice carrying ticks, which in turn was correlated with mouse density at a site. Other host present at our study sites in this region (e.g., the deer mouse, shrews, voles, sciurids, or ground-dwelling birds) are also competent reservoirs of *B. burgdorferi* (see Ostfeld 2011) but are likely less efficient in transmitting *B. burgdorferi* to feeding larval ticks than *P. leucopus* (Rand et al. 1993). Both our results and previous work (reviewed by Ostfeld 2011) thus point to the importance of the white-footed mouse in determining the presence of *B. burgdorferi*.

### The drivers: climate warming

The key potential role of climate warming on the northern expansion of Lyme borreliosis in Europe and North-America has been noted (e.g., Lindgren et al. 2000). The average temperature during the growing season has increased by 0.8°C over the last four decades in Montérégie (Almaraz et al. 2008). This recent increase in average annual temperature has created more favorable conditions for the establishment of tick and white-footed mouse populations and contributed to the northern expansion of the distribution range of these two species (Brownstein et al. 2005; Ogden et al. 2006; Roy-Dufresne et al. 2013). Migratory birds are breeding further north in Québec each year (DesGranges and Morneau 2010), dropping ticks that face new environmental conditions compared with their site of origin. Ambient temperature is limiting the distribution of ticks by affecting their rate of development from one life stage to the next (Ogden et al. 2005). In particular, the temperature during the summer must be warm enough for the ticks to complete their lifecycle (Ogden et al. 2005), and cold temperature during the winter may preclude the survival of overwintering ticks in the far north (but see Brunner et al. 2012). Likewise, the presence of the white-footed mouse is favored by short winter and warm temperature and its range is rapidly shifting poleward (Roy-Dufresne et al. 2013). Overall, there is thus an increasing body of empirical evidence to support the hypothesis that climate warming is a key driver of Lyme disease emergence, acting upon many levels of the transmission cycle of the disease. Although rates of northward range expansion of high risk zones for *B. burgdorferi* in eastern Canada may be limited by the rates at which *P. leucopus* can expand its range with climate change, it is also apparent from our study that relatively modest changes in northward range expansion will likely be accompanied by very much greater expansion of the range of the rodent eastwards and westward (Figure S2). Clearly, in large areas of southern Québec, winters are currently too cold or long for *P. leucopus* survival; however, only small warming changes will be needed for a wide geographic area to become suitable for this species. This has considerable public health significance because, as for most of Canada, most of the human population in Québec lives within a few hundred kilometers of the US border and along the shores of the St Lawrence River. Therefore changes in Lyme disease risk in this region will likely have the greatest influence on Lyme disease incidence in Canada.

### The drivers: habitat change

Coincident with recent climate warming, is the intensification of agriculture and deforestation that has led to the fragmentation of the landscapes and a mosaic of agriculture lands and forested patches. In Southern Québec, the degree of forest habitat lost is high, with a 70% decrease in old growth forest in the Montérégie alone over the last 70 years (Wampach 1988). While climate, especially temperature, plays a key role in limiting the distribution of the tick, we found that landscape variables (including both land use, patch area and connectivity measures) were a significant predictor of the abundance of the tick. The connectivity between habitat patches in a fragmented landscape has been shown to affect tick densities, which are higher in this type of mosaic landscape than in more homogeneous and continuous forested landscapes (e.g., Barbour and Fish 1993; Brownstein et al. 2005). A higher tick density in fragmented areas (Killilea et al. 2008) presumably reflects a higher abundance of hosts for feeding and reproduction in these habitats (e.g., Nupp and Swihart 1996; Allan et al. 2003; Anderson and Meikle 2006); the white-footed mouse benefits from higher edge habitats in small forest fragments (Wilder et al. 2005). Small forest patches are often surrounded by less suitable habitats which may hinder dispersion and promote genetic and morphological differentiation between isolated populations (Klein and Cameron 2012; Munshi-South 2012; Ledevin and Millien 2013; Rogic et al. 2013; R. R. Marrotte, A. Gonzalez, and V. Millien unpublished data). There is thus empirical evidence that features of the landscape such as less favorable habitats or physical barriers (e.g., rivers and roads) are limiting dispersal of the white-footed mouse. Habitat fragmentation is thus expected to affect the pattern of the spread of Lyme disease in Southern Québec, as it is limiting the distribution of both the tick and the mouse. However, we found that habitat-related variables were much less influential than climatic-related ones in predicting the presence of *B. burgdorferi*.

### Other factors and the limits of our approach

While our study provides robust evidence for the combined effects of the expansion of both the tick and the mouse on the rate of emergence of Lyme disease, our approach has several limitations.

First, our risk index at the regional-Québec scale was estimated from the potential distribution of the tick and mouse, which is limited by climatic factors. Yet, the realized distribution of the tick and the mouse is likely constrained by a number of other interacting factors, in addition to climatic ones (Elith and Leathwick 2009; Araujo and Peterson 2012); these include landscape structure and the existence of geographic barriers (Bennie et al. 2013; Reino et al. 2013; Rioux-Paquette et al. 2014), the interaction with coexisting species (e.g. competition, Araujo and Peterson 2012), and dispersal behavior (Elith and Leathwick 2009). At the landscape scale, we found that the landscape was limiting the distribution of both the white-footed mouse and the black-legged tick, but to a lesser extent than climate variables. Overall, there is a considerable body of evidence that climate plays a major role in determining species distribution (Araujo and Peterson 2012), an hypothesis confirmed by our results.

Second, the climate model we used for the back-legged tick was a simple model linking tick abundance and temperature. Temperature is a key factor limiting the current distribution, establishment rate and future northern expansion of tick populations. However, other climatic factors are also important at limiting the distribution of the black-legged tick. Ticks, for example, are sensitive to desiccation while questing and require a minimum level of humidity. On the other hand, an excess of rain will hinder activity levels of the ticks, and decrease their chances of obtaining the blood meal they require to transition to their next life stage. Leighton et al. (2012) found that temperature was the main factor contributing to the establishment of tick population, but other variables such as rainfall and elevation were also included in their model. These conclusions are in agreement with the conclusion of Ogden et al. (2005) who found that when taken alone, temperature likely over-predicts the actual suitable range of black-legged ticks in Southern Canada.

Third, as noted in Paull et al. (2012), climate-driven changes in disease ecology are constrained by a number of other underlying and interacting factors (Ogden et al. 2013). For example, in her study of tick populations in Europe, Randolph (2008) found that *Ixodes ricinus* quested earlier in response to climate warming. However, this resulted in a mismatch between the timing of questing of larvae and the rise of small mammal host abundance, which in turn resulted in an increased mortality rate in ticks, and a decreased prevalence of tick-borne encephalitis (TBE) in their hosts (Randolph 2008). In this case, climate warming was not linked to an increase risk in human cases of TBE.

Here, we argue that while the presence of ticks is essential for *B. burgdorferi* transmission, ticks alone cannot predict the occurrence of *B. burgdorferi*, and that the white-footed mouse is a key element driving the distribution of the pathogen in this area of rapid invasion. However, other hosts that we did not consider may be equally important. The main host for the adult, reproductive stage of the tick, is the white-tailed deer (*Odocoileus virginianus*). There has been an increase in abundance of the white-tailed deer in North America during the 20th century (Ostfeld 2011), and in southern Québec, white-tailed deer populations have exceeded historical records of abundance in recent years (Huot and Lebel 2012).

Finally, we hypothesized that the magnitude of the effects of climate on the tick and mouse distribution does not change with time. Yet, this remains to be confirmed, especially in the context of range expansion when local adaptation of marginal populations may occur (e.g., Hill et al. 2011). Using neutral genetic markers and landmark data from the skull, we detected a strong geographical differentiation of populations of the white-footed mouse across forest patches within our study area (Ledevin and Millien 2013; Rogic et al. 2013). Interestingly, the patterns of genetic and morphological differentiation matched each other, hinting to a possible rapid—over the last few decades—evolution of populations isolated in favorable forested habitat within a landscape of less favorable habitat. With that scenario, each isolated population of white-footed mouse would have adapted to the local biotic and abiotic conditions, which could potentially alter its relationship with the black-legged tick (e.g. through behavioral adaptation) and ultimately the efficiency of the transmission cycle of *B. burgdorferi* locally. Alternatively, the local genetic and phenotypic differentiation we observed in the white-footed mouse could be the result of multiple independent founder events within an heterogeneous landscape, where each propagule and founding population would only carry a subset of the gene pool and phenotypic traits found in the original core population (Bell 2013). In this case as well, both the relation between the white-footed mouse and the black-legged tick and the transmission rate of *B. burgdorferi* may vary across populations. A change in disease spread within these isolated populations could occur for example in response to a change in the genetic diversity of the white-footed mouse populations (e.g., King and Lively 2012). Predictions about the future risk of spread of Lyme disease in Southern Québec should consider the potential for evolutionary change in the multiple species involved in the transmission of *B. burgdorferi*, and how evolution can alter the consequences of shifts in distributions of these species in response to environmental changes on disease occurrence (e.g., Hendry et al. 2011; Lankau et al. 2011). An effort to integrate both ecological and evolutionary responses of hosts, vectors and their pathogens will improve management of Lyme disease; a conclusion that likely extends more generally to the effects of diseases on wildlife (e.g., Vander Wal et al. 2014).

## Conclusion

Our study aimed at combining both field and modeling approaches to better understand the pattern and rate of Lyme disease spread in Southern Québec in the next few decades. Here, we have identified a strong association of *B. burgdorferi* with the presence of *P. leucopus* in a zone of emergence of Lyme disease risk. Clearly, the presence of *P. leucopus* is not a sine qua non for *B. burgdorferi* invasion; this bacterium is a host generalist (Hanincova et al., 2006), it is emerging in a number of locations in Manitoba where *P. leucopus* does not occur (and *P. maniculatus* is the dominant rodent species: L. R. Lindsay, unpublished data), and the bacterium was identified at one of our sites where *P. leucopus* was not found. Nevertheless, here *B. burgdorferi* was most often found in locations where both *I. scapularis* and *P. leucopus* occurred, which suggests that the presence of *P. leucopus* facilitates *B. burgdorferi* invasion. Communities where *P. leucopus* occur are likely producing more efficient *B. burgdorferi* transmission cycles, resulting in higher infection prevalence in small mammals and ticks. These *P. leucopus*-rich communities are therefore hot spots for Lyme disease risk, and we predict that the geographic scope of these hot spots will spread north with climate change. While our risk maps are helpful for identifying areas of highest risk for Lyme disease, our work also provides a baseline for comparison to future studies of surveillance and thus helps at quantifying the rate of emergence of Lyme disease in the region. We recommend that combined studies of the distribution of ticks and white-footed mouse are conducted to assist decision-making on managing Lyme disease risk in Québec. Such studies will facilitate the identification of current and future areas of high risk of occurrence of *B. burgdorferi* in the region. While the overall prevalence of *B. burgdorferi* host-seeking ticks in Southern Québec is still low (~13%, this study; Ogden et al. 2010) in comparison with the rates of >20% typically observed in the United States (Gatewood et al. 2009), the risk of acquiring Lyme disease in Québec is now real and growing (Bourre-Tessier et al. 2011). Future work should be designed to integrate data on human recreational and professional activities and link the environmental risk (i.e., the presence of *B. burgdorferi*) with Lyme disease risk in humans (Medlock and Jameson 2010).

## Data archiving statement

Data for this study are available in the supporting information online.

## Supporting Information

**Figure S1.** Current (A) and future (B) predicted abundance (the maximum annual number of feeding female ticks at equilibrium) of the black-legged tick, based on DD > 0.

**Figure S2.** Probability of presence for the current (A) and future (B) projected distribution of the white-footed mouse, based on climatic variables.

**Figure S3.** Factor map of the principal component analysis performed on landscape variables.

**Table S1.** Field data for the 34 field sites.
