# Supplementary figures and images for: Climate change and habitat fragmentation drive the occurrence of Borrelia burgdorferi, the agent of Lyme disease, at the northeastern limit of its distribution
Source: Evol Appl. 2014 May 7;7(7):750–64. doi: 10.1111/eva.12165 (PMC4227856; doi:10.1111/eva.12165)

## Slide 1
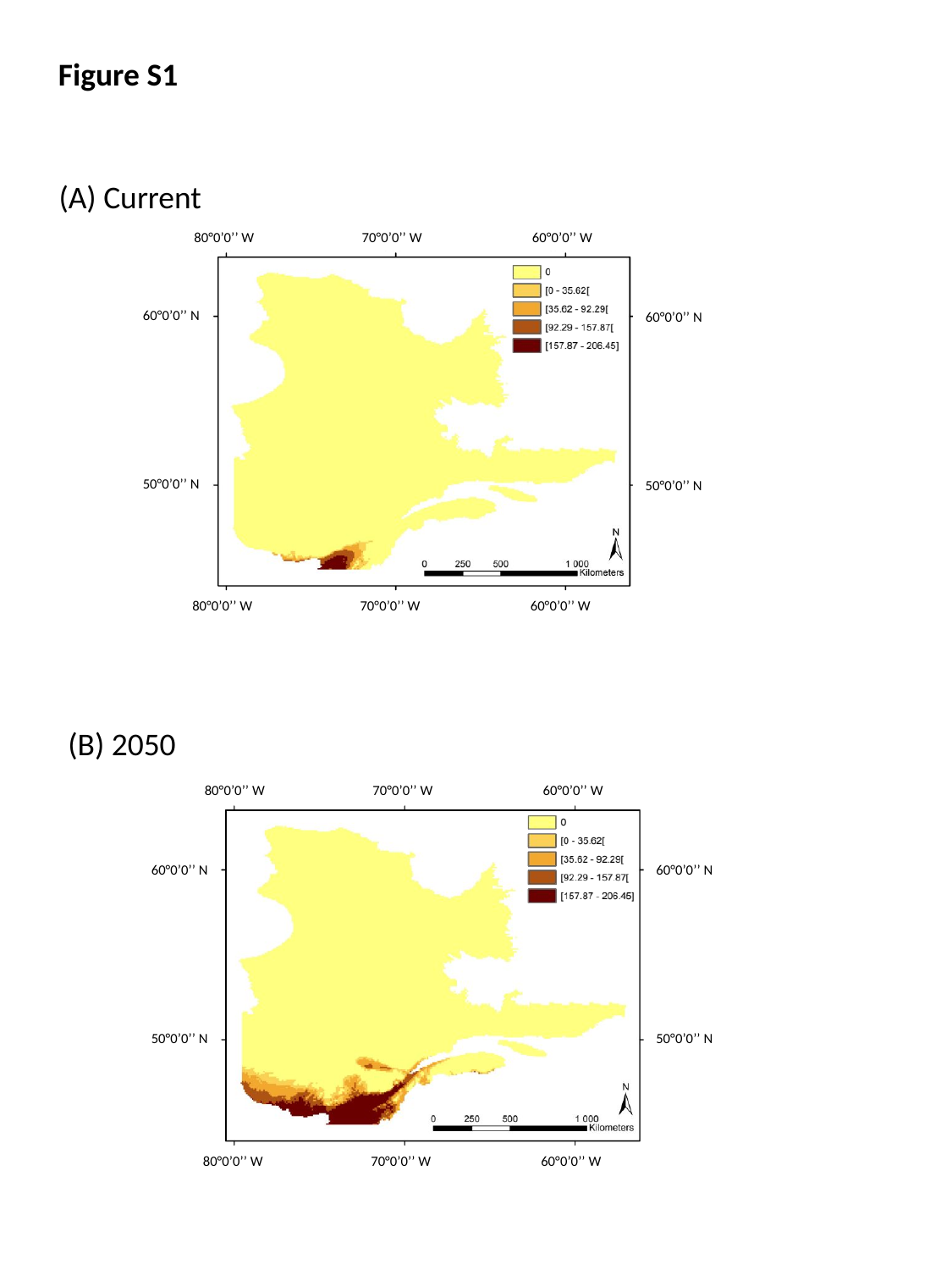

Figure S1
(A) Current
80°0’0’’ W
70°0’0’’ W
60°0’0’’ W
60°0’0’’ N
60°0’0’’ N
50°0’0’’ N
50°0’0’’ N
80°0’0’’ W
70°0’0’’ W
60°0’0’’ W
(B) 2050
80°0’0’’ W
70°0’0’’ W
60°0’0’’ W
60°0’0’’ N
60°0’0’’ N
50°0’0’’ N
50°0’0’’ N
80°0’0’’ W
70°0’0’’ W
60°0’0’’ W

Supplement: Supplementary file 1 — Figure S1. Current (A) and future (B) predicted abundance (the maximum annual number of feeding female ticks at equilibrium) of the black-legged tick, based on DD > 0. [file eva0007-0750-sd1.pptx]

## Slide 1
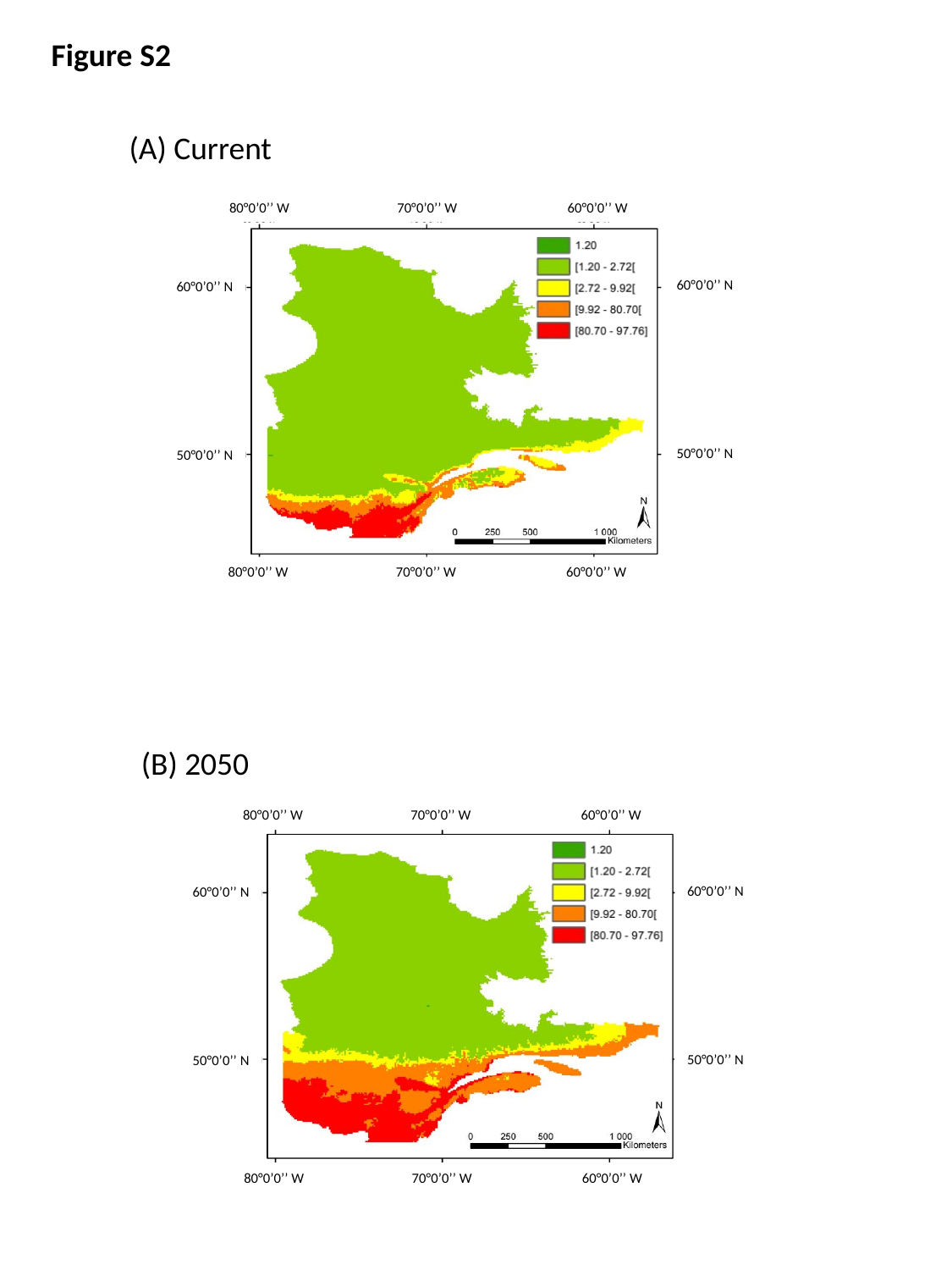

Figure S2
(A) Current
80°0’0’’ W
70°0’0’’ W
60°0’0’’ W
60°0’0’’ N
60°0’0’’ N
50°0’0’’ N
50°0’0’’ N
80°0’0’’ W
70°0’0’’ W
60°0’0’’ W
 (B) 2050
80°0’0’’ W
70°0’0’’ W
60°0’0’’ W
60°0’0’’ N
60°0’0’’ N
50°0’0’’ N
50°0’0’’ N
80°0’0’’ W
70°0’0’’ W
60°0’0’’ W

Supplement: Supplementary file 2 — Figure S2. Probability of presence for the current (A) and future (B) projected distribution of the white-footed mouse, based on climatic variables. [file eva0007-0750-sd2.pptx]

## Slide 1
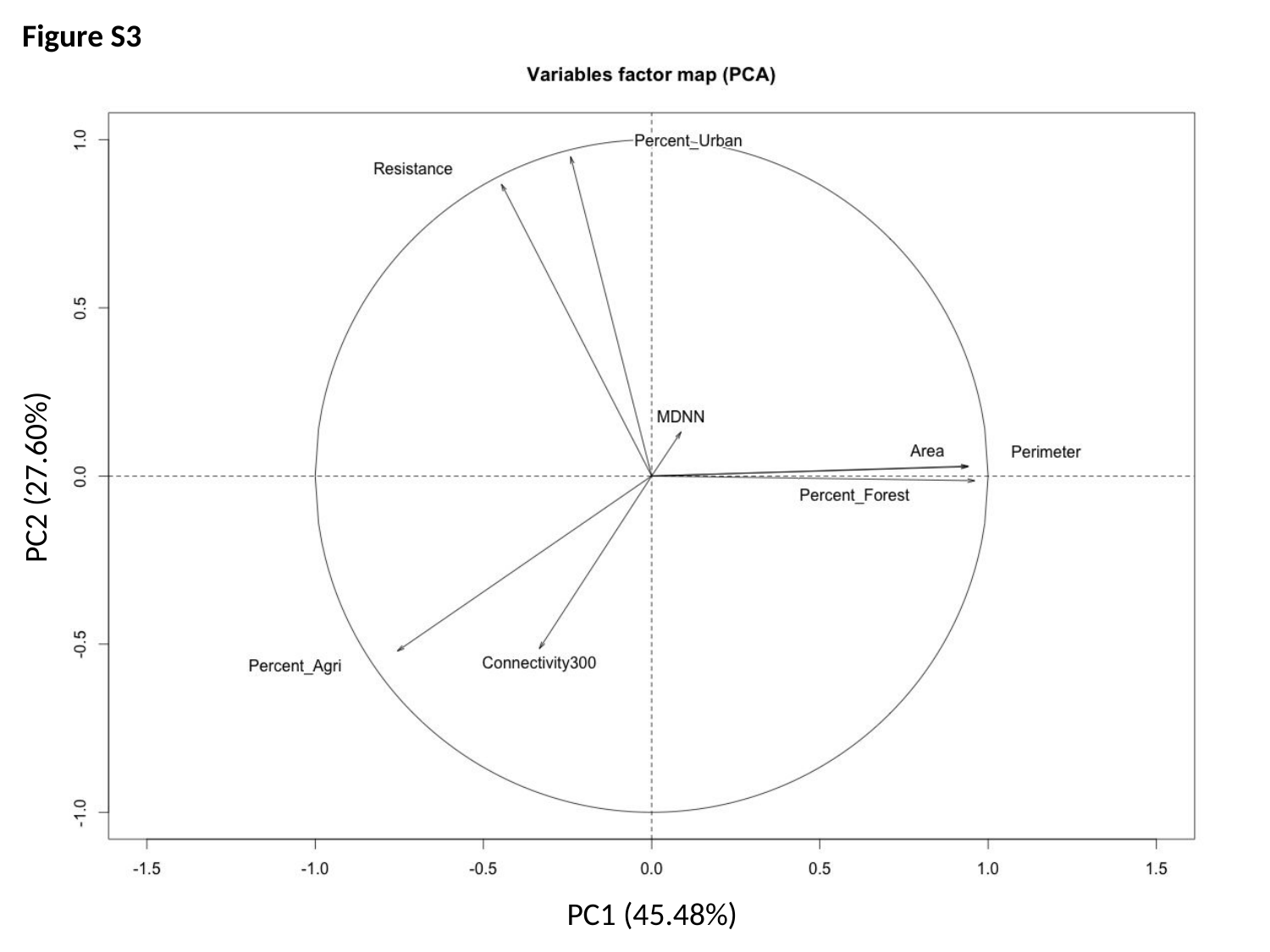

Figure S3
PC2 (27.60%)
PC1 (45.48%)

Supplement: Supplementary file 3 — Figure S3. Factor map of the principal component analysis performed on landscape variables. [file eva0007-0750-sd3.pptx]
