# Supplementary material for: Climate change and habitat fragmentation drive the occurrence of Borrelia burgdorferi, the agent of Lyme disease, at the northeastern limit of its distribution
Source: Evol Appl. 2014 May 7;7(7):750–64. doi: 10.1111/eva.12165 (PMC4227856; doi:10.1111/eva.12165)
Supplement: Supplementary file 5 [file eva0007-0750-sd5.docx]

**Supporting Information**

Table  S1 Field data for the 34 field sites; SP : Species richness at a site, #Mam: number of small mammals sampled (Pl : *Peromyscus leucopus*, Pm : *Peromyscus maniculatus*, Mg : *Myodes gapperi*, Bl : *Blarina brevicauda*, So : *Sorex* sp. Ni : *Napaeozapus insignis*), *Ixodes scapularis*: number of larvae feeding on small mammals (adjusted by trapping effort), number of larvae, nymphs and adults sampled questing in the vegetation and total number of ticks sampled (all stages), Bb : *Borrelia burgdorferi* status (0: absent, 1: present).

| **Site** | **Latitude** | **Longitude** | **SP** | **#Mam** | **Pl** | **Pm** | **Mg** | **Bl** | **So** | **Ni** | **FeedingL** | **QuestingT** | **TotalT** | **Bb** |
| --- | --- | --- | --- | --- | --- | --- | --- | --- | --- | --- | --- | --- | --- | --- |
| 1 | -72.25 | 45.20 | 4 | 15 | 0 | 7 | 3 | 2 | 0 | 3 | 2.5 | 2 | 4.5 | 0 |
| 2 | -73.01 | 45.31 | 4 | 10 | 3 | 2 | 3 | 0 | 0 | 2 | 134 | 370 | 504 | 1 |
| 3 | -73.23 | 45.48 | 3 | 16 | 13 | 1 | 0 | 2 | 0 | 0 | 0.2 | 0 | 0.2 | 0 |
| 4 | -72.02 | 45.81 | 2 | 4 | 0 | 1 | 3 | 0 | 0 | 0 | 0 | 7 | 7 | 0 |
| 5 | -73.08 | 45.46 | 1 | 5 | 5 | 0 | 0 | 0 | 0 | 0 | 0 | 3 | 3 | 0 |
| 6 | -72.55 | 45.86 | 2 | 13 | 5 | 0 | 8 | 0 | 0 | 0 | 2 | 5 | 7 | 1 |
| 7 | -73.20 | 45.38 | 3 | 36 | 27 | 0 | 0 | 7 | 0 | 2 | 5.125 | 57 | 62.25 | 1 |
| 8 | -73.18 | 45.13 | 3 | 8 | 5 | 1 | 2 | 0 | 0 | 0 | 3 | 132 | 137 | 1 |
| 9 | -73.31 | 45.49 | 3 | 58 | 31 | 0 | 0 | 2 | 25 | 0 | 7.125 | 13 | 20.125 | 1 |
| 10 | -73.20 | 45.51 | 1 | 35 | 35 | 0 | 0 | 0 | 0 | 0 | 0.625 | 0 | 0.625 | 0 |
| 11 | -73.17 | 45.47 | 3 | 24 | 19 | 0 | 0 | 1 | 4 | 0 | 0 | 2 | 2.2 | 0 |
| 12 | -73.46 | 45.81 | 1 | 5 | 5 | 0 | 0 | 0 | 0 | 0 | 0 | 6 | 6 | 1 |
| 13 | -72.41 | 45.73 | 2 | 3 | 2 | 0 | 0 | 1 | 0 | 0 | 0 | 0 | 0 | 0 |
| 14 | -72.90 | 45.44 | 4 | 46 | 34 | 1 | 5 | 5 | 1 | 0 | 3.75 | 18 | 22 | 0 |
| 15 | -73.46 | 45.54 | 1 | 10 | 10 | 0 | 0 | 0 | 0 | 0 | 5 | 15 | 20 | 0 |
| 16 | -72.14 | 46.22 | 1 | 3 | 0 | 3 | 0 | 0 | 0 | 0 | 0 | 1 | 1 | 0 |
| 17 | -73.05 | 45.47 | 4 | 26 | 18 | 0 | 1 | 6 | 1 | 0 | 0 | 1 | 1 | 0 |
| 18 | -72.85 | 45.46 | 3 | 28 | 13 | 13 | 0 | 2 | 0 | 0 | 0.286 | 9 | 9.286 | 0 |
| 19 | -72.58 | 46.17 | 3 | 21 | 0 | 4 | 16 | 1 | 0 | 0 | 0 | 2 | 2.5 | 0 |
| 20 | -73.29 | 45.06 | 3 | 9 | 7 | 0 | 1 | 0 | 1 | 0 | 0 | 18 | 21 | 1 |
| 21 | -73.06 | 45.42 | 3 | 41 | 35 | 0 | 0 | 5 | 1 | 0 | 0.222 | 13 | 13.444 | 0 |
| 22 | -72.60 | 45.43 | 2 | 8 | 0 | 7 | 0 | 1 | 0 | 0 | 3 | 9 | 12 | 0 |
| 23 | -72.19 | 45.32 | 2 | 9 | 0 | 8 | 0 | 1 | 0 | 0 | 0 | 0 | 0 | 0 |
| 24 | -73.28 | 45.98 | 4 | 12 | 0 | 9 | 2 | 1 | 0 | 0 | 0 | 28 | 28 | 0 |
| 25 | -72.14 | 45.97 | 4 | 8 | 0 | 2 | 5 | 0 | 0 | 1 | 0 | 25 | 25 | 0 |
| 26 | -73.93 | 45.21 | 2 | 8 | 6 | 0 | 0 | 2 | 0 | 0 | 2 | 9 | 11 | 0 |
| 27 | -73.46 | 45.24 | 1 | 2 | 2 | 0 | 0 | 0 | 0 | 0 | 0 | 5 | 5 | 0 |
| 28 | -73.01 | 45.77 | 3 | 9 | 7 | 0 | 1 | 1 | 0 | 0 | 0 | 0 | 0 | 0 |
| 29 | -72.74 | 45.65 | 2 | 16 | 14 | 0 | 0 | 2 | 0 | 0 | 0 | 1 | 1 | 0 |
| 30 | -74.34 | 45.24 | 2 | 11 | 10 | 0 | 0 | 1 | 0 | 0 | 0 | 15 | 15 | 0 |
| 31 | -73.17 | 45.87 | 1 | 5 | 4 | 0 | 0 | 0 | 0 | 1 | 0 | 1 | 3 | 0 |
| 32 | -73.32 | 45.12 | 1 | 4 | 0 | 0 | 4 | 0 | 0 | 0 | 0 | 35 | 37 | 1 |
| 33 | -74.07 | 45.23 | 1 | 2 | 2 | 0 | 0 | 0 | 0 | 0 | 3 | 17 | 20 | 1 |
| 34 | -74.00 | 45.98 | 3 | 10 | 0 | 1 | 8 | 0 | 0 | 1 | 0 | 0 | 0 | 0 |

**Figure S1.** Current (A) and future (B) predicted abundance (the maximum annual number of feeding female ticks at equilibrium) of the black-legged tick, based on DD > 0. The projected distribution for 2050 was modeled with a change in climate under a combination of A1b, A2 and B1 greenhouse gas emissions scenarios from the IPCC (Nakicenovic et al 2000) (WGS 1984 World Mercator). Model from Ogden et al. (2005).

**Figure S2.** Probability of presence for the current (A) and future (B) projected distribution of the white-footed mouse, based on climatic variables. The projected distribution for 2050 was modeled with a change in climate under a combination of A1b, A2 and B1 greenhouse gas emissions scenarios from the IPCC (Nakicenovic et al 2000) (WGS 1984 World Mercator). Model from Roy-Dufresne et al. (2013).

**Figure S3.** Factor map of the principal component analysis performed on landscape variables. Percent_Forest: proportion of forest, Percent_Agri: proportion of agriculture, Percent_Urban: proportion of urban habitat, Area: forest patch area, Perimeter: forest patch perimeter, MDNN: Minimum distance to the nearest neighbour, Resistance: resistance and Connectivity300: Connectivity (at a 300 m resolution). The first two principal components explained 73.08% of the variance.
